# Layer-by-Layer (LbL) Surface Augmented Modification of Poly(Styrene/Divinylbenzene)High Internal Phase Emulsion for Carbon Dioxide Capture

**DOI:** 10.3390/polym13142247

**Published:** 2021-07-09

**Authors:** Muhammad Imran Azman, Jirasuta Chungprempree, Jitima Preechawong, Pornsri Sapsrithong, Manit Nithitanakul

**Affiliations:** 1 The Petroleum and Petrochemical College, Chulalongkorn University, Chula Soi 12, Wangmai Pathumwan, Bangkok 10330, Thailand; imran.espana@gmail.com (M.I.A.); jirasuta.chung@gmail.com (J.C.); jitima.pre@gmail.com (J.P.); 2 Division of Polymer Engineering Technology, Department of Mechanical Engineering Technology, College of Industrial Technology, King Mongkut’s University of Technology North Bangkok, Bangkok 10800, Thailand; pornsri.s@cit.kmutnb.ac.th

**Keywords:** CO_2_ adsorption, layer-by-layer (LbL) technique, polyHIPE

## Abstract

In this study, we used amines electrolyte solution with layer-by-layer (LbL) technique to modify and increase the CO_2_ adsorption capacity of highly porous polymer from high internal phase emulsion template polymer. This perspective presents the extraordinary versatility of emulsion templating polymerization, which has emerged with the growing numbers of HIPE systems and modification. In this study, we used polyHIPE prepared from styrene (S) and divinylbenzene (DVB) with varying ratios; 80:20, 20:80, and 0:100 to improve the surface area, thermal properties, and mechanical properties of the materials. Furthermore, the surface of the polyHIPE was modified by LbL technique to increase the adsorption efficiency. This technique consisted of two main layers, the primary layer of poly(diallyldimethylammonium chloride) (PDADMAC) and polystyrene sulfonate (PSS) and the secondary layer, which was the CO_2_ adsorbing layer, of polyethylene imine (PEI) or tetraethylene pentamine (TEPA). Poly(S/DVB)HIPE modified by PEI terminated as the secondary coating showed the highest CO_2_ adsorption capacity, with up to 42% (from 0.71 to 1.01 mmol/g). The amine-multilayered modified material still possessed an open cell structure, since the solution did not block the pore structure of the poly(S/DVB)HIPE and was suitable for being used as an adsorbent in adsorption technology.

## 1. Introduction

With the rapid growth of many industries around the world, an excessive amount of greenhouse gas has been released into the air, especially as emission from industry [1]. For instance, the concentration of carbon dioxide has increased dramatically from 280 part per million (ppm) in pre-industrial time to 400 ppm in 2013, representing a 43% increase [2]. All greenhouse gases adversely affect human health and many technologies have been modified to reduce the amount of toxic gas. Technologies have also been developed to capture carbon dioxide followed by storage or utilization, and these technologies include absorption technology [3], adsorption technology [4], and membrane separation [5,6]. The solution absorption is one of the most cost-effective technologies for CO_2_ capture. Actual commercial solution absorption consisted of organic solvents and green solvents (ionic liquids, deep eutectic solvents, and liquid polymers) for CO_2_ capture [7]. However, aqueous amine absorption has some problems, namely corrosion, oxidation degradation and using a high amount of energy to regenerate [8]. Due to this, most of the applications for CO_2_ are adsorption-related high superabsorbent material that have been produced through the generation of microporosity and/or mesoporosity material [9]. Scientists also developed adsorbent for a carbon dioxide capture process by using activated carbon from biomass product (seaweed and lawn grass) as solid adsorbent [10,11]. In addition, cryogenic distillation has also been widely used for CO_2_ absorption. Nevertheless, there were several drawbacks for cryogenic distillation, which were stated as being costly, especially for extracting all the water [12]. 

To overcome these problems, porous polymer prepared by high internal phase emulsion (HIPE) was studied for the CO_2_ adsorption application. Polymer from high internal phase emulsion (polyHIPE) has been developed as it contains highly interconnected open-cell structure with porosities, surface area, and high thermal degradation [13]. It could be prepared from an external phase (oil phase) and internal phase (aqueous phase) under mechanical stirring. Typically, the total volume of the aqueous phase exceeds 74 to 90% [14] and the material has many good properties, such as low density (less than 0.03 g/cm^2^), a high interconnected open cell structure, and a high surface area (up to 700 m^2^/g). PolyHIPE is based on the polymerization of monomers and crosslinking comonomer within the organic (continuous) phase of water-in-oil (w/o). The organic phase is less than 26% of the total volume generally consisting of monomer, surfactant, and solvent [15]. In addition, the capacity of CO_2_ adsorption could be further improved by introducing amine groups such as tetraethylenepentamine (TEPA)/diethanolamine (DEA) [16], polyethyleneimine (PEI) [17,18], diisopropanalamine (DIPA) [19], and piperazine (PZ) [20] onto the substrates. However, an impregnated technique inside porous can aggregate, leading to pore plugging [18] and thus eliminating the pore plugging, which occurs when using the layer-by-layer (LbL) technique.

Layer-by-layer polyelectrolyte multilayers (LbL) technique for the formation of thin film is employed for its simplicity in methodology. The concept was popularized in the 1990s by Gero Decher’s group at the Universite Louis Pasteur and CNRS in France. Polycation (e.g., polyethylenimine (PEI), tetraethylenepentamine (TEPA)), and polyanion (e.g., polystyrene sulfonate (PSS) and polyacrylic acid (PAA)) on a polyHIPE were studied. These amine-multilayered sorbents had much faster CO_2_ adsorption and desorption rates compared to sorbents prepared using the current PEI-impregnation approach [18]. The polyHIPEs were further improved by introducing LbL technique with the introduction of the ionic groups from amination methods in order to enhance the capacity of the CO_2_ absorption [21]. In this study, polyHIPE was prepared from divinylbenzene (DVB) and styrene (S) using water in oil system. Then, CO_2_ adsorbing solutions were introduced onto the polyHIPE using LbL technique. This technique consisted of two main layers: the primary layer, consisting of six layers of PDADMAC and PSS, which were polycation and polyanionic layers and the secondary layer, which was the CO_2_ adsorbing layers of polyethylenimine (PEI) and tetraethylenepentamine (TEPA) layers. Finally, polyHIPEs were characterized by scanning electron microscope (SEM), Autosorb-1MP (Quanta chrome), Universal testing machine (Lloyd), differential thermal analysis (TG-DTA), and gas chromatography with a thermal conductivity detector (GC-TCD). With a functional styrene monomer that has been synthesized to be a mesoporous material, which ostensibly would be more compatible for the adsorption and release of the CO_2_, it was obvious that the product gave a very promising outcomes for the reusability in comparison with recent popular research such as cryogenic distillation, hydrogel membrane, and monolith gel [22].

## 2. Materials and Methods

### 2.1. Material

Divinylbezene (DVB) and styrene (S) were purchased from Merck (Darmstadt, Germany) and Sigma-Aldrich (St. Louis, MO, USA), respectively. Surfactant, sorbitant monolate (Span 80), dodecylben-zolsolfonic acid, sodium salt (DDBSS), were purchased from Sigma-Aldrich chemical (Missouri, USA) and cetyltrimethylammoium bromide (CTAB) was supplied from Fluka Chemie (Buchs, Switzerland). The initiator and stabilizer were potassium persulphate (K_2_S_2_O_8_, purity ≥ 98% (RT), Fluka Chemie, Buchs, Switzerland) and calcium chloride (CaCl_2_, purity ≥ 97% (KT), Fluka Chemie, Buchs, Switzerland). Toluene (T) was supplied by RCI Lab scan (Bangkok, Thailand). Polystyrenesulfonate (PSS, sodium salt, MW 70,000), poly(diallyldimethylammonium chloride) (PDADMAC, MW 350,000), polyethyleneimine (PEI, MW 2000), and tetraethylenepentamine (TEPA, MW 198) were purchased from Sigma-Aldrich (Missouri, USA). All the chemicals were used as received.

### 2.2. Poly(Styrene/Divinylbenzene)HIPE Polymerization

**Scheme 1:**
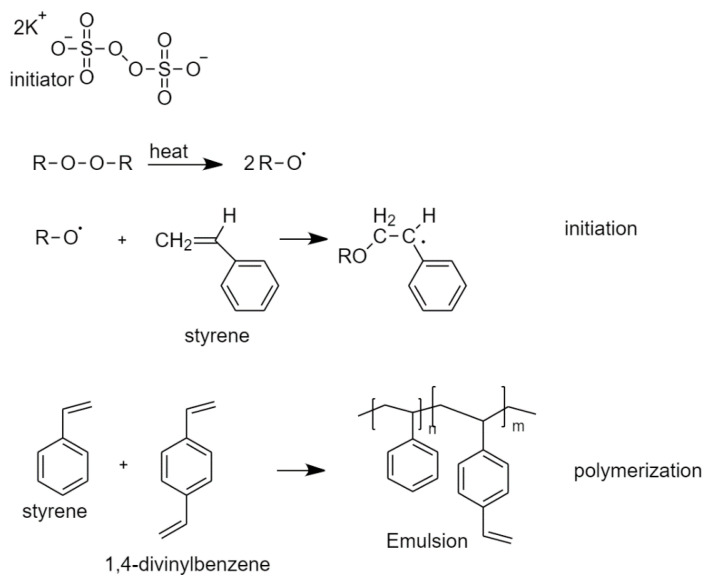
The process mechanism of poly(S/DVB)HIPE polymerization.

PolyHIPEs were prepared from styrene and divinylbenzene monomers. The process mechanism of poly(S/DVB)HIPE is presented in Scheme 1. The ratios of styrene to divinylbenzene were 80:20, 20:80, and 0:100. The polyHIPEs were prepared with a mix surfactant (Span 80, CTAB, DDBSS, 6.3 wt%, 0.3 wt%, 0.4 wt%) and 5 mL of solvent as toluene, which were mixed together. The organic phase and the aqueous phase contained 90 mL of deionized water, 0.2 g of potassium persulphate as an initiator, and 1 g of stabilizer salt (CaCl_2_). After preparing the two phases, the aqueous phase to the organic phase was slowly dropped with mechanical stirring at 360 rpm. Next, the obtained emulsion was poured into a glass mold and polymerization in a water bath at 60 °C for 48 h. Then, it was removed from the glass mold and dried in a conventional oven at 60 °C for 24 h. After polymerization, the unreacted chemical was extracted from polyHIPE sample with 2-propanol using a soxhlet apparatus for 6 h. Finally, the material was returned to dry in an oven at 60 °C until a constant weight. 

### 2.3. Surface Modification of Poly(S/DVB)HIPEs

**Figure 1:**
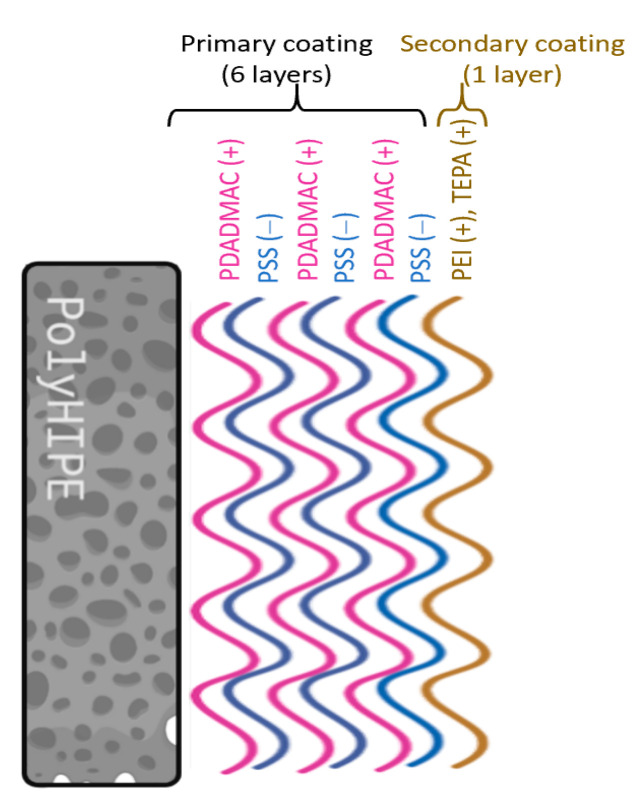
Layer-by-layer polyelectrolyte membrane fabricated on polyHIPEs surface.

The layer-by-layer polyelectrolyte multilayers (LbL) technique was used to modify the polyHIPE surface. Poly(S/DVB)HIPE was cut into 2 cm thick sample. Then, a vacuum pump was applied to run the coating solutions through poly(S/DVB)HIPE. The LbL technique is composed of two coatings: the primary coating and the secondary coating. For the primary coating, a positively charged solution of PDADMAC was run through poly(S/DVB)HIPEs for 2 min, then the poly(S/DVB)HIPE was rinsed with DI water 2 times. Next, a negatively charged solution of PSS was run through poly(S/DVB)HIPE for 2 min, then the poly(S/DVB)HIPE was rinsed with DI water. The deposition of the primary layer contained six layers. For the secondary coating, tetraethylenepentamine (TEPA) or polyethylenimine (PEI) polymer solution were used as the termination coating, as shown in Figure 1. Finally, the sample was dried at room temperature for 24 h.

**Scheme 2:**
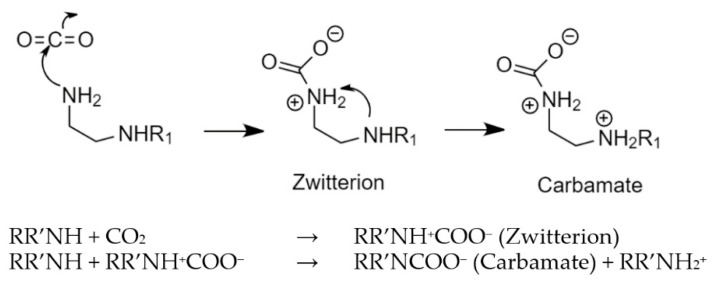
The CO_2_ adsorption mechanism of primary and secondary amine groups via the carbamate mechanism.

The use of polyHIPE modified with primary (TEPA) and secondary (PEI) amines is the economic option. The adsorption of CO_2_ on amine-functionalized modified polyHIPE proceeds predominantly by the carbamate mechanism, as shown in Scheme 2.

The amount of CO_2_ gas adsorbed by poly(S/DVB)HIPEs was determined using a gas chromatography instrument. The samples were loaded into a sample tube 2 × 25 cm. Before carrying out the adsorption studies, the poly(S/DVB)HIPEs sample in the reactor was pretreated with N_2_ (80 mL/min). Then, the gas was switched to 15 vol% of CO_2_ (15 mL/min) at room temperature and desorption was applied with N_2_ (80 mL/min). Finally, adsorption capacity of poly(S/DVB)HIPEs was calculated by Equation (1) below.

(1) $$Q_{\mathrm{ads}}=\frac{{\mathrm{FC}}_{\mathrm{in}}t_{\mathrm{st}}}{M},$$

where Q_ads_ dynamic adsorption capacity, mmol CO_2_/g, F = total flow rate, mol/min, C_in_ = the concentration of CO_2_ entering the reactor, vol%, M = the weight of the adsorbent, g, and t_st_ = the stoichiometric time corresponding to CO_2_ stoichiometric adsorption capacity, min.

### 2.4. Poly(S/DVB)HIPEs Characterization

Average pore size and surface morphological of each sample were observed by scanning electron microscope (SEM). The specimens were cut into small pieces and coated with platinum under vacuum before analysis. The surface area was calculated by BET equation by a Quantachrome Autosorb-1MP obtained from the N_2_ adsorption-desorption isotherms at –196 °C. The samples were degassed at 100 °C for 12 h in a vacuum furnace. For mechanical properties, they were investigated by Lloyd Universal testing machine in a cylinder shape sample at the diameter of 2.54 cm and times 2.54 cm of its height. A speed of 0.127 cm/min and 500 Newton load cells were used for all measurements. Thermogravimetric analysis (TGA) was performed to measure the thermal stability of the poly(S/DVB)HIPEs under N_2_ flow of 100 mL/min. The samples were fragmented into small pieces weighing about 2 to 5 mg. Then the samples were heated from 30 °C to 800 °C with a constant heating rate of 10 °C/min. The degradation temperature was determined from the weight loss (%) vs. temperature curve. UV-Vis Spectroscopy was used to monitor the amine coating of layer-by-layer process. Amine functionalization in all modified polyHIPEs with the amino group were investigated by CHN elemental analysis. 

## 3. Results

### 3.1. Characterization of Poly(S/DVB)HIPEs

**Figure 2:**
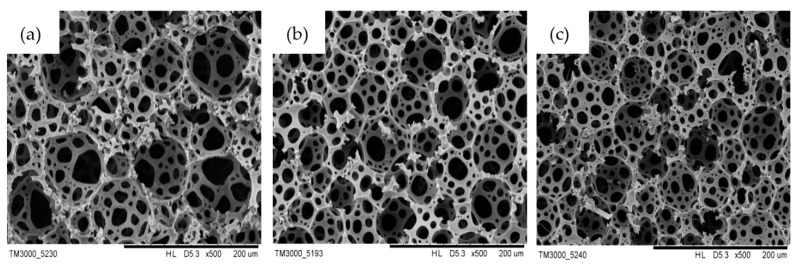
Scanning electron micrographs of different ratio S/DVB of poly(S/DVB)HIPE: (**a**) 80:20, (**b**) 20:80, and (**c**) 0:100.

Typically, the structure of polyHIPEs are mesoporous polymers with interconnecting pores or windows [23]. Figure 2 provides information about the morphology of poly(S/DVB)HIPE using SEM micrographs with a magnification of ×500, prepared by a three component surfactant (SPAN80, DDBSS, and CTAB) and difference amounts of styrene and divinylbenzene in the emulsion systems. The pore size diameters of poly(S/DVB)HIPE were found to decrease cell size when increasing the amount of divinylbenzene. For 20 vol%, 80 vol%, and 100 vol% DVB, the average pore diameters of poly(S/DVB)HIPE structure were 79.4 μm, 58.8 µm, and 41.2 μm, respectively. As a results, the decrease of the average pore diameters of poly(S/DVB)HIPE were affected by the crosslinking comonomer (DVB). The poly(S/DVB)HIPE system was incorporated more easily when the system increased the divinylbenzene content, which the crosslink network formed earlier [24].

Surface areas of poly(S/DVB)HIPEs prepared by using three difference ratios of S/DVB i.e., 80:20, 20:80, and 0:100 were measured by an Autosorb-1MP machine. The result showed that poly(S/DVB)HIPEs had a surface area between 22 and 363 m^2^/g. The surface area of poly(S/DVB)HIPEs tended to increase with the increased amount of DVB in the system, as shown in Table 1, due to the ability of a high degree of crosslinking (DVB), making the structure more stable [25].

The mechanical properties of poly(S/DVB)HIPE were studied using the LLOYD universal testing machine. In Table 1, information on compressive strength and Young’s modulus of poly(S/DVB)HIPE were given. When increasing the divinylbenzene content, the compressive strength of the polyHIPE was found to increase from 0.13 to 0.30 MPa. Young’s modulus of sample was increased from 1.79 to 5.41 MPa. The highest compressive strength and Young’s modulus of poly(S/DVB)HIPE was a S/DVB ratio of 0:100, mainly because a large amount of crosslinking gave a stronger structure, so when increasing the amount of DVB in the system, poly(S/DVB)HIPE became more stable than others [25].

Thermal properties of poly(S/DVB)HIPE were investigated by TG analysis. The decomposition temperature (T_d_) and residue yield of poly(S/DVB)HIPEs were also listed in Table 1. The result showed that T_d_ and the residue yield of the samples increased with the increased concentration of divinylbenzene. Due to the high concentration of DVB, it led to a high degree of crosslinking, so the structure of poly(S/DVB)HIPE became more stable [25]. Generally, material used for CO_2_ capture are exposed to temperatures in excess of 300 °C, so the poly(S/DVB)HIPEs prepared in this study would be suitable for this application [26]. 

### 3.2. Surface Modification of Poly(S/DVB)HIPE

**Figure 3:**
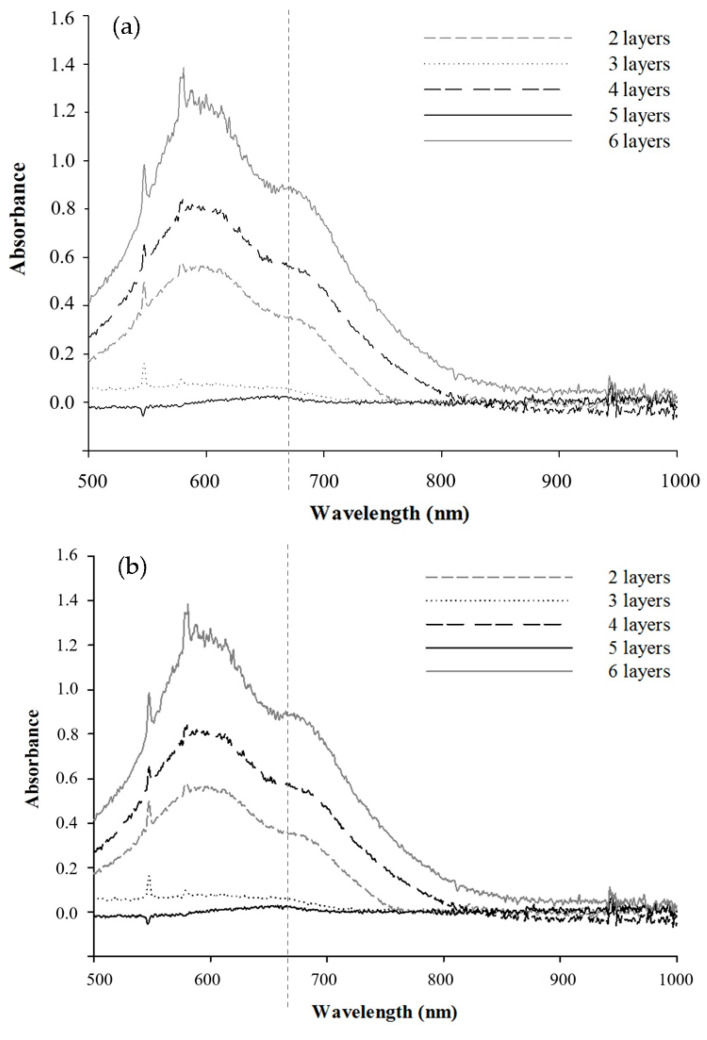
Absorbance-number of layer curves for PDAD/PSS deposited 1.0 M NaCl: (**a**) top surface of poly(S/DVB)HIPE; (**b**) cross section of poly(S/DVB)HIPE.

**Figure 4:**
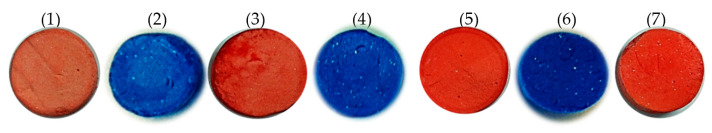
Photograph of cross-section of poly(S/DVB)HIPE modified surface: primary coating contain PDADMAC/PSS; PDADMAC on top of surface (**1**), (**3**), and (**5**); PSS on top of surface (**2**), (**4**), and (**6**) and secondary coating contain TEPA or PEI (**7**) as termination coating.

In order to confirm the primary coating layer (consisting of three bilayers of alternating layers of two polyions, PDADMAC(+)/PSS(−)), UV–Vis spectrophotometer was employed. The number of increased layer of the polyelectrolytes for each layer within poly(S/DVB)HIPE was monitored by a UV–Vis spectrophotometer with the absorption of light at 670 nm. The intensity of absorbance of the top surface and cross section of poly(S/DVB)HIPE increased with the increase in the number of layers from two to six layers, as shown in Figure 3a,b. Six layers of PDADMAC/PSS were sufficient to successfully modified the surface of the poly(S/DVB)HIPE. Figure 4 showed that congo red can react with cation (PDADMAC or amine coating) on the surface (1, 3, 5, and 7 layers). Methylene blue can react with anion (PSS) on the surface (2, 4, and 6 layers). The homogenous coating inside and outside poly(S/DVB)HIPE was confirmed (Figure 4). The results of the photographs of poly(S/DVB)HIPE with dye are illustrated. It was observed that the depth of color increases with the number of layers (see Figure 4).

**Figure 5:**
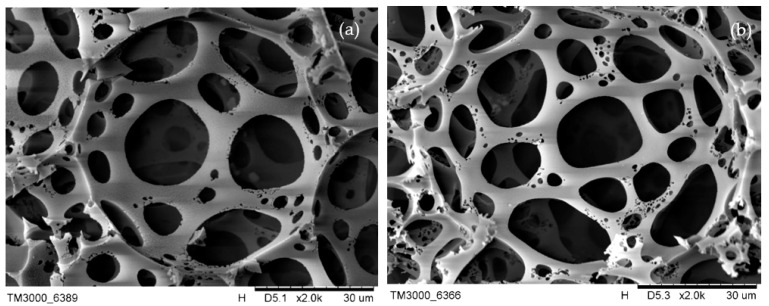
Scanning electron micrographs of poly(S/DVB)HIPE: (**a**) unmodified S/DVB 80:20 (×2000); (**b**) modified S/DVB 80:20 (×2000).

The study was followed by morphology of unmodified and modified poly(S/DVB)HIPEs. As shown in Figure 5, the micrographs showed similar images before and after the modification with amine solutions. It can be concluded that amine solution coated on poly(S/DVB)HIPEs had little influence on the pore structure, as they neither ruptured or blocked the porosities of the polyHIPEs. 

### 3.3. CO_2_ Adsorption Capacities

**Figure 6:**
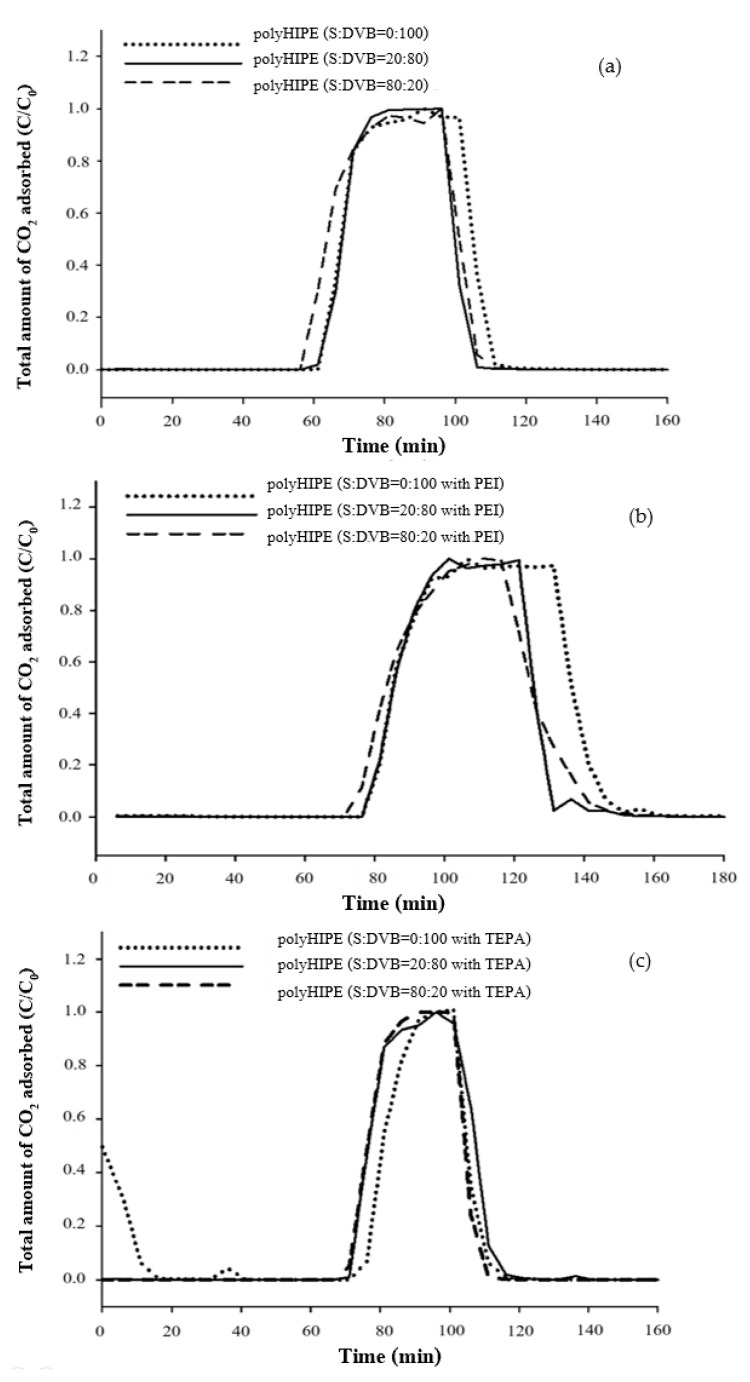
Comparison of the CO_2_ breakthrough curve of (**a**) poly(S/DVB)HIPEs filled with different ratio of S/DVB, (**b**) modified poly(S/DVB)HIPEs with PEI and (**c**) modified poly(S/DVB) HIPEs with TEPA.

The CO_2_ adsorption of neat poly(S/DVB)HIPE and LbL amine-modified poly(S/DVB)HIPE were analyzed. Poly(S/DVB)HIPE was modified by amine solution of polyethylenimine (PEI) or tetraethylenepentamine (TEPA). CO_2_ adsorption system was carried out using a mixture gas 15 vol% of CO_2_ in N_2_ with a pressure of 30 psi at room temperature. Figure 6a–c shows the CO_2_ breakthrough curve of unmodified and amine-modified poly(S/DVB)HIPEs. Unmodified poly(S/DVB)HIPE at 100 vol% DVB has slightly higher capacity compared to (S/DVB 20:80) and (S/DVB 80:20) due to the resulting high surface area, so poly(S/DVB)HIPEs had higher physical adsorption. Compared to unmodified poly(S/DVB) HIPE, amine modified poly(S/DVB)HIPE illustrated the higher CO_2_ adsorption rate due to the ability of chemical adsorption and physical adsorption, as both adsorptions lead to higher CO_2_ adsorption capacity. 

Table 2 provides data on the CO_2_ adsorption capacity of poly(S/DVB)HIPE between unmodified and amine-modified poly(S/DVB)HIPE. Significantly, CO_2_ adsorption of S/DVB 0:100 modified by PEI had the highest adsorption capacity of 1.01 mmol/g. In addition, elemental analysis results in Table 2 illustrate the difference in the percent of amine by CHN analysis due to the effect of adhesive amine on different surface areas of polyHIPE. After the secondary coating (consisting of a layer of polycations, which are TEPA or PEI) were applied, the element of CHN analysis were used to confirm the amount of carbon, hydrogen, and nitrogen of the modified poly(S/DVB)HIPEs. From Table 2, results indicate mass fraction percentage of amine by CHN elemental analysis with the different ratios of DVB and styrene. It is shown that moderate amounts, 0.52% and 0.59%, of nitrogen were observed in the 20:80 and 80:20 ratios of styrene and divinylbenzene, respectively. The highest mass fraction of nitrogen was observed in polyHIPE with the 0:100 ratio, and this is because the highest abundancy of pores was coated with amine modification [27].

Several kinds of technologies have been employed to reduce the amount of CO_2_ in the atmosphere by CO_2_ adsorption. The CO_2_ adsorption performance of different materials were compared and outlined in Table 3. From the table, it was found that the modified polyHIPE with PEI by LbL technique gave a high CO_2_ adsorption capability at 1.01 ± 0.27 mmol/g. 

## 4. Conclusions

Poly(S/DVB)HIPEs were successfully prepared from high internal phase emulsions technique and improved the property using difference ratio of DVB in organic phase. The obtained poly(S/DVB)HIPEs have an open cell structure with an average diameter of 79.4, 58.8, and 41.2 µm and a surface area of 22, 189, and 363 m^2^/g for S/DVB at 80:20, 20:80, and 0:100, respectively. Moreover, increasing the concentration of divinylbenzene, the thermal degradation temperature of the poly(S/DVB)HIPE increased from 372 to 440 °C and the compressive strength increased from 0.13 to 0.30 MPa. Subsequently, poly(S/DVB)HIPE became more stable. In addition, poly(S/DVB)HIPEs were completely modified on the surface with the layer-by-layer polyelectrolyte multilayer (LbL) technique. Poly(S/DVB)HIPE modified by primary coating at three bilayers and PEI terminated as the secondary coating with high amounts of DVB (S/DVB: 0:100) in monomer ratio is the best surface modification to increase the CO_2_ adsorption capacity up to 42% (from 0.71 to 1.01 mmol/g), when compared with the unmodified poly(S/DVB)HIPE. Compared to unmodified poly(S/DVB)HIPE, PEI modified poly(S/DVB)HIPE illustrated the highest CO_2_ adsorption capacity due to the ability of chemical and physical adsorption. Results from modified poly(S/DVB)HIPE suggested that it has high adsorption capacity, can easily desorb, and has low energy of desorption and low cost of adsorption-desorption materials.

## Figures and Tables

**Table 1:** Physical, mechanical, and thermal properties of poly(S/DVB)HIPEs prepared using a different S/DVB ratio.

Poly(S/DVB) HIPE	Surface Area (m^2^/g)	Compressive Strength (MPa)	Young’s Modulus (MPa)	T_d_ (°C)	Residue Yield (%)
80:20	22.39 ± 10.30	0.13 ± 0.02	1.79 ± 0.44	373.79	8.18
20:80	189.40 ± 16.14	0.29 ± 0.09	3.59 ± 1.12	432.80	24.81
0:100	363.06 ± 149.77	0.30 ± 0.03	5.41 ± 1.29	440.98	32.51

**Table 2:** CO_2_ adsorption of poly(S/DVB)HIPE between the unmodified and amine-modified surface.

(S/DVB) Ratio	Mass Fraction of Nitrogen (%)		Adsorption Capacity (mmol/g)		
	Modified polyHIPE with PEI	Modified polyHIPE with TEPA	Unmodified Surface	Modified polyHIPE with PEI	Modified polyHIPE with TEPA
0:100	0.81	1.54	0.71 ± 0.19	1.01 ± 0.27	0.72 ± 0.11
20:80	0.59	0.52	0.64 ± 0.12	0.82 ± 0.62	0.72 ± 0.13
80:20	0.52	0.56	0.63 ± 0.15	0.68 ± 0.16	0.64 ± 0.04

**Table 3:** CO_2_ adsorption with different materials.

Materials	CO_2_ Adsorption Capacity (mmol/g)	Ref.
polyHIPE	0.71 ± 0.19	This paper
Modified polyHIPE with PEI	1.01 ± 0.27	This paper
Modified polyHIPE with TEPA	0.72 ± 0.11	This paper
Natural bentonite clay	0.15 mmol/g	[28]
Acid- natural bentonite clay	0.38 mmol/g	[28]
Activated carbon prepared from lawn grass	0.12 mmol/g	[11]
Ionic liquids	4.72 mol of CO_2_/mol of solvent	[7]
Deep eutectic solvents	4.29 g CO_2_ per g of solvent	[7]
Liquid polymers	1.36 mol of CO_2_/mol of solvent	[7]

## References

[1] Songolzadeh M., Soleimani M., Takht Ravanchi M., Songolzadeh R. (2014). Carbon Dioxide Separation From Flue Gases: A Technological, Review Emphasizing Reduction in Greenhouse Gas Emissions. Sci. World J..

[2] Medlyn B., De Kauwe M. (2013). Carbon dioxide and water use in forests. Nature.

[3] Hamborg E.S., Derks P.W.J., van Elk E.P., Versteeg G.F. (2011). Carbon dioxide removal by alkanolamines in aqueous organic solvents. A method for enhancing the desorption process. Energy Procedia.

[4] Yu C.-H., Huang C.-H., Tan C.-S. (2012). A Review of CO _2_ Capture by Absorption and Adsorption. Aerosol Air Qual. Res..

[5] Norahim N., Yaisanga P., Faungnawakij K., Charinpanitkul T., Klaysom C. (2018). Recent Membrane Developments for CO_2_ Separation and Capture. Chem. Eng. Technol..

[6] Prasetya N., Himma N.F., Sutrisna P.D., Wenten I.G., Ladewig B.P. (2020). A review on emerging organic-containing microporous material membranes for carbon capture and separation. Chem. Eng. J..

[7] Krishnan A., Gopinath K.P., Vo D.-V.N., Malolan R., Nagarajan V.M., Arun J. (2020). Ionic liquids, deep eutectic solvents and liquid polymers as green solvents in carbon capture technologies: A review. Environ. Chem. Lett..

[8] Islam M., Yusoff R., Ali B. (2010). Degradation studies of amines and alkanolamines during CO_2_ absorption and stripping system. Eng. E Trans..

[9] Israel S., Gurevitch I., Silverstein M.S. (2015). Carbons with a hierarchical porous structure through the pyrolysis of hypercrosslinked emulsion-templated polymers. Polymer.

[10] Laurens L.M.L., Lane M., Nelson R.S. (2020). Sustainable Seaweed Biotechnology Solutions for Carbon Capture, Composition, and Deconstruction. Trends Biotechnol..

[11] Aslam Z., Anait U., Abbas A., Ihsanullah I., Irshad U., Mahmood N. (2020). Adsorption of carbon dioxide onto activated carbon prepared from lawn grass. Biomass Convers. Biorefinery.

[12] Barbieri G., Brunetti A., Scura F., Drioli E. (2011). CO_2_ Separation by Membrane Technologies: Applications and Potentialities. Chem. Eng. Trans..

[13] Pakeyangkoon P., Magaraphan R., Malakul P., Nithitanakul M. (2008). Effect of Soxhlet Extraction and Surfactant System on Morphology and Properties of Poly(DVB)PolyHIPE. Macromol. Symp..

[14] Cameron N. (2005). High Internal Phase Emulsion Templating as a Route to Well-defined Porous Polymers. Polymer.

[15] Silverstein M.S. (2014). Emulsion-templated porous polymers: A retrospective perspective. Polymer.

[16] Yue M., Sun L.-B., Cao Y., Wang Z., Wang Y., Yu Q., Zhu J. (2008). Promoting the CO _2_ adsorption in the amine-containing SBA15 by hydroxyl group. Microporous Mesoporous Mater..

[17] Martín A., García R., Karaman D.S., Rosenholm J. (2014). Polyethyleneimine-functionalized large pore ordered silica materials for poorly water-soluble drug delivery. J. Mater. Sci..

[18] Jiang B., Kish V., Fauth D.J., Gray M.L., Pennline H.W., Li B. (2011). Performance of amine-multilayered solid sorbents for CO_2_ removal: Effect of fabrication variables. Int. J. Greenh. Gas Control.

[19] Plaza M.G., Pevida C., Arias B., Fermoso J., Arenillas A., Rubiera F., Pis J.J. (2008). Application of thermogravimetric analysis to the evaluation of aminated solid sorbents for CO_2_ capture. J. Therm. Anal. Calorim..

[20] Saiwan C., Muchan P., deMontigny D., Tontiwachwutikul P. (2014). New Poly(Vinylbenzylchloride/Divinylbenzene) Adsorbent for Carbon Dioxide Adsorption. II. Effect of Amine Functionalization. Energy Procedia.

[21] Hou J., Cao S., Wu Y., Liang F., Sun Y., Lin Z., Sun L. (2017). Simultaneously efficient light absorption and charge transport of phosphate and oxygen-vacancy confined in bismuth tungstate atomic layers triggering robust solar CO_2_ reduction. Nano Energy.

[22] Wright A.J., Main M.J., Cooper N.J., Blight B.A., Holder S.J. (2017). Poly High Internal Phase Emulsion for the Immobilization of Chemical Warfare Agents. ACS Appl. Mater. Interfaces.

[23] Barbetta A., Cameron N.R. (2004). Morphology and Surface Area of Emulsion-Derived (PolyHIPE) Solid Foams Prepared with Oil-Phase Soluble Porogenic Solvents:  Three-Component Surfactant System. Macromolecules.

[24] Erbay E., Okay O. (1999). Pore memory of macroporous styrene-divinylbenzene copolymers. J. Appl. Polym. Sci..

[25] Jin J.M., Yang S.H., Han S.T., Choe S.J. (2006). Highly crosslinked poly(acrylamide-co-divinylbenzene) microspheres by precipitation polymerization. J. Ind. Eng. Chem..

[26] D’Alessandro D.M., Smit B., Long J.R. (2010). Carbon Dioxide Capture: Prospects for New Materials. Angew. Chem. Int. Ed..

[27] Lei L., Zhang Q., Shi S., Zhu S. (2017). Highly Porous Poly(high internal phase emulsion) Membranes with “Open-Cell” Structure and CO_2_-Switchable Wettability Used for Controlled Oil/Water Separation. Langmuir.

[28] Volzone C. (2007). Retention of pollutant gases: Comparison between clay minerals and their modified products. Appl. Clay Sci..

